# rs1990622 variant associates with Alzheimer’s disease and regulates *TMEM106B* expression in human brain tissues

**DOI:** 10.1186/s12916-020-01883-5

**Published:** 2021-01-19

**Authors:** Yang Hu, Jing-yi Sun, Yan Zhang, Haihua Zhang, Shan Gao, Tao Wang, Zhifa Han, Longcai Wang, Bao-liang Sun, Guiyou Liu

**Affiliations:** 1 School of Life Science and Technology, Harbin Institute of Technology, Harbin, 150080 China; 2 Shandong Provincial Hospital, Shandong First Medical University & Shandong Academy of Medical Sciences, Jinan, 250021 China; 3 Department of Pathology, The Affiliated Hospital of Weifang Medical University, Weifang, 261053 China; 4 Beijing Institute for Brain Disorders, Laboratory of Brain Disorders, Ministry of Science and Technology, Collaborative Innovation Center for Brain Disorders, Capital Medical University, Beijing, 100069 China; 5 Academy for Advanced Interdisciplinary Studies, Peking University, Beijing, China; 6 Chinese Institute for Brain Research, Beijing, China; 7 School of Medicine, School of Pharmaceutical Sciences, THU-PKU Center for Life Sciences, Tsinghua University, Beijing, China; 8 State Key Laboratory of Medical Molecular Biology, Institute of Basic Medical Sciences, Chinese Academy of Medical Sciences, Beijing, China; 9 Department of Pathophysiology, Peking Union Medical College, Beijing, China; 10 Department of Anesthesiology, The Affiliated Hospital of Weifang Medical University, Weifang, 261053 China; 11 Key Laboratory of Cerebral Microcirculation in Universities of Shandong; Department of Neurology, Second Affiliated Hospital; Shandong First Medical University & Shandong Academy of Medical Sciences, Taian, 271000 Shandong China; 12 National Engineering Laboratory of Internet Medical Diagnosis and Treatment Technology, Xuanwu Hospital, Capital Medical University, Beijing, 100053 China; 13 Department of Neurology, Xuanwu Hospital, Capital Medical University, Beijing, 100053 China

**Keywords:** *TMEM106B*, Alzheimer’s disease, Genome-wide association study, Neurological diseases, eQTLs

## Abstract

**Background:**

It has been well established that the *TMEM106B* gene rs1990622 variant was a frontotemporal dementia (FTD) risk factor. Until recently, growing evidence highlights the role of *TMEM106B* in Alzheimer’s disease (AD). However, it remains largely unclear about the role of rs1990622 variant in AD.

**Methods:**

Here, we conducted comprehensive analyses including genetic association study, gene expression analysis, eQTLs analysis, and colocalization analysis. In stage 1, we conducted a genetic association analysis of rs1990622 using large-scale genome-wide association study (GWAS) datasets from International Genomics of Alzheimer’s Project (21,982 AD and 41,944 cognitively normal controls) and UK Biobank (314,278 participants). In stage 2, we performed a gene expression analysis of *TMEM106B* in 49 different human tissues using the gene expression data in GTEx. In stage 3, we performed an expression quantitative trait loci (eQTLs) analysis using multiple datasets from UKBEC, GTEx, and Mayo RNAseq Study. In stage 4, we performed a colocalization analysis to provide evidence of the AD GWAS and eQTLs pair influencing both AD and the *TMEM106B* expression at a particular region.

**Results:**

We found (1) rs1990622 variant T allele contributed to AD risk. A sex-specific analysis in UK Biobank further indicated that rs1990622 T allele only contributed to increased AD risk in females, but not in males; (2) *TMEM106B* showed different expression in different human brain tissues especially high expression in cerebellum; (3) rs1990622 variant could regulate the expression of *TMEM106B* in human brain tissues, which vary considerably in different disease statuses, the mean ages at death, the percents of females, and the different descents of the selected donors; (4) colocalization analysis provided suggestive evidence that the same variant contributed to AD risk and *TMEM106B* expression in cerebellum.

**Conclusion:**

Our comprehensive analyses highlighted the role of FTD rs1990622 variant in AD risk. This cross-disease approach may delineate disease-specific and common features, which will be important for both diagnostic and therapeutic development purposes. Meanwhile, these findings highlight the importance to better understand *TMEM106B* function and dysfunction in the context of normal aging and neurodegenerative diseases.

## Background

TMEM106B is a lysosomal protein and belongs to the TMEM106 family of proteins with relatively unknown function [1, 2]. In 2010, a common *TMEM106B* genetic variant rs1990622 (T>C) was first identified to be associated with frontotemporal dementia (FTD) risk (OR = 1.64 for T allele, allele frequency = 0.679, and *P* = 1.08E−11) [3]. Since the identification of rs1990622 as an FTD risk variant, kinds of studies have been conducted to understand the role of this non-coding mutation, which is located downstream 6.9 kb 3′ of *TMEM106B* [4–6].

Li and colleagues conducted a cell type quantitative trait loci (cQTL) analysis using 2008 brain samples derived from 1536 unique individuals including 640 AD, 488 cognitively healthy controls, 11 FTD, 75 progressive supranuclear palsy, 28 pathological aging, 189 schizophrenia, 30 bipolar disorders, and 75 individuals with other unknown dementia or no diagnosis information [4]. Interestingly, Li and colleagues identified *TMEM106B* variant rs1990621 C allele, which is in high linkage disequilibrium with rs1990622 variant T allele, to be significantly associated with the reduced neuronal proportion [4].

Until recently, Yang and colleagues conducted a module quantitative trait loci (modQTL) analysis to identify genetic variants regulating the average expression of the genes found in the gene co-expression modules [5]. Interestingly, they found rs1990622 variant to show a significant modQTL effect, and highlighted *TMEM106B* as key aging human brain transcriptome regulator [5]. Meanwhile, Yang and colleagues identified that myelination and lysosomal genes regulated by *TMEM106B* could connect amyloid-β (Aβ) and TAR DNA-binding protein 43 kDa (TDP-43) [5]. It is known that increased Aβ is a key Alzheimer’s disease (AD) neuropathology. Hence, Yang and colleagues provided important findings about the key pathogenic link between AD and TDP-43 proteinopathy [5]. However, Yang and colleagues did not directly evaluate the association between rs1990622 variant and AD risk. Until now, it remains unclear whether rs1990622 variant is associated with AD risk, although a lack of significant association between rs1990622 variant and AD risk [7]. We think that this may be caused by inadequate sample sizes (300 AD cases and 137 controls) [7], and large-scale samples are needed.

Meanwhile, Yang and colleagues conducted an expression quantitative trait loci (eQTLs) analysis of rs1990622 variant using 494 human prefrontal cortex samples from the Religious Orders Study and Memory and Aging Project (ROSMAP) [8]. They found that rs1990622 variant T allele could significantly increase *TMEM106B* expression (β = 0.067, and *P* = 5.90E−05) [5]. However, gene expression analysis did not support the increased *TMEM106B* expression in human brain tissues. Satoh et al. evaluated the expression levels of TMEM106B in AD and control frontal cortex and the hippocampus tissues [9]. They selected 6 sporadic AD patients and 13 controls including 4 normal subjects without neurological disease, 3 patients with sporadic Parkinson’s disease, 4 patients with sporadic amyotrophic lateral sclerosis, and 2 patients with sporadic multiple system atrophy [9]. They demonstrated that both the mRNA and protein levels of TMEM106B were significantly reduced in AD brains compared control brains [9].

In discussion, Yang and colleagues concluded that the pre-existing neurodegenerative proteinopathies were not necessary to drive the association between rs1990622 variant and *TMEM106B* transcriptome dysregulation [5]. However, recent findings from other *TMEM106B* variants did not support this conclusion. Ren and colleagues conducted a stratification analysis and highlighted more pronounced effects of *TMEM106B* rs3173615 variant on the transcriptome in neurodegenerative diseases than in healthy controls [6]. Li and colleagues conducted a stratification analysis and found that *TMEM106B* rs1990621 variant could regulate the neuronal proportion in AD cases, other neurodegenerative diseases, elderly cognitively healthy controls, but not young controls [4]. All these findings indicated that the link between *TMEM106B* haplotype and transcriptome dysregulation is context dependent [4, 6]. Importantly, rs1990622 variant is in high linkage disequilibrium with rs3173615 (*r*^2^ = 0.98 and *D′* = 1) and rs1990621 (*r*^2^ = 0.99 and *D′* = 1). Hence, we consider that the association between rs1990622 variant and *TMEM106B* transcriptome dysregulation may also be context dependent.

Until now, large-scale AD genome-wide association study (GWAS) datasets and large-scale eQTLs datasets in both the neuropathologically normal individuals and neurological disease individuals have provided strong support to answer these concerns [10, 11]. Here, we conducted comprehensive analyses using publicly available datasets. In stage 1, we conducted a genetic association analysis to investigate the effect of rs1990622 variant on AD risk using multiple large-scale GWAS datasets. In stage 2, we performed a gene expression analysis of *TMEM106B* in 49 different human tissues. In stage 3, we performed an eQTLs analysis to evaluate the effect of rs1990622 variant on *TMEM106B* expression in multiple human brain tissues with different disease statuses. In stage 4, we performed a colocalization analysis to provide evidence of the AD GWAS and eQTLs pair influencing both AD and the *TMEM106B* expression at a particular region.

## Methods

### AD GWAS datasets

We selected two independent large-scale AD GWAS dataset resources from International Genomics of Alzheimer’s Project (IGAP) stage 1 [10] and UK Biobank [11]. The IGAP stage 1 consisted of 21,982 AD and 41,944 cognitively normal controls of European descent [10]. These individuals are from four consortia including Alzheimer Disease Genetics Consortium (ADGC), Cohorts for Heart and Aging Research in Genomic Epidemiology Consortium (CHARGE), the European Alzheimer’s Disease Initiative (EADI), and Genetic and Environmental Risk in AD/Defining Genetic, Polygenic and Environmental Risk for Alzheimer’s Disease Consortium (GERAD/PERADES) [10]. AD patients are diagnosed using the NINCDS-ADRDA criteria or DSM-IV guidelines [10]. In UK Biobank, AD GWAS was conducted in 314,278 participants including 27,696 maternal cases and 14,338 paternal cases [11]. Meanwhile, UK Biobank also included two sex-specific AD GWAS datasets including one AD GWAS dataset in males diagnosed by paternal history of AD (14,338 cases and 245,941 controls) and one AD GWAS dataset in females diagnosed by maternal history of AD (27,696 cases and 260,980 controls) [11].

### eQTLs datasets from neuropathologically normal and disease individuals

We selected two independent eQTLs dataset resources from the neuropathologically normal individuals. The first resource is from the UK Brain Expression Consortium (UKBEC), which included 134 neuropathologically normal individuals of European descent [12]. The UKBEC consisted of 10 eQTLs datasets in 10 brain regions including cerebellar cortex, frontal cortex, hippocampus medulla, occipital cortex, putamen, substantia nigra, temporal cortex, thalamus, and intralobular white matter [12]. The second resource is from the Genotype-Tissue Expression (GTEx, version 8) [13]. The GTEx included 13 eQTLs datasets in 13 brain tissues (amygdala/amygdalae, anterior cingulate cortex, caudate basal ganglia, cerebellar hemisphere, cerebellum, cortex, frontal cortex, hippocampus, hypothalamus, nucleus accumbens, putamen, spinal cord, and substantia nigra) [13]. About 99% of the donors of these brain tissues are neuropathologically normal individuals, and 1% of the donors of these brain tissues died of neurological diseases (1.3% in age 20–39 and 1.2% in age 60–71) [13]. Recent studies have provided more detailed information about these datasets [14–19].

The eQTLs dataset resource in neurological disease individuals is from the Mayo RNAseq Study [20]. Mayo eQTLs datasets included 773 brain samples, which could be further divided into 197 AD cerebellar samples, 202 AD temporal cortex samples, 177 non-AD cerebellar samples, and 197 non-AD temporal cortex samples [20]. The non-AD samples have several brain pathologies including PSP, LBD, corticobasal degeneration, FTD, multiple system atrophy, and vascular dementia [20].

The main demographic profiles of the selected eQTLs datasets are provided in Table 1. In brief, the selected donors in UKBEC are of European descent with mean age at death 59 and 26% of these donors were female. The selected donors in GTEx are of multiple descents including European (85.3%), African (12.3%), Asian (1.4%), and Hispanic or Latino (1.9%), with mean age at death 55, and 33% of these donors were female. The selected donors in Mayo are of European descent with mean age at death 74 in AD and 72 in non-AD, and 51–53% of these donors were female in AD and 36–40% of these donors were female in non-AD. In order to further compare the findings from ROSMAP, we also included the demographic profiles of the ROSMAP eQTLs datasets, as described in Table 1. In ROSMAP, the selected donors are of European descent with mean age at death 88 and 62% of these donors were female. Thirty-nine percent of these donors were diagnosed with clinical AD, and 58% were pathological AD.

**Table 1:** Demographic profiles of the selected eQTLs datasets

Dataset	Diagnosis	Donors #	Mean age at death	Descent	Females %
UKBEC	Neuropathologically normal	134	59	European	26
GTEx	Neuropathologically normal	838	55	European (85.3%)	33
				African (12.3%)	
				Asian (1.4%)	
				Hispanic or Latino (1.9%)	
Mayo	AD (cerebellar)	197	74	European	51
Mayo	Non-AD (cerebellar)	177	72	European	36
Mayo	AD (temporal cortex)	202	74	European	53
Mayo	Non-AD (temporal cortex)	197	72	European	40
ROSMAP	39% clinical AD	494	88	European	62
	58% pathological AD				

### Genetic association analysis

For the genetic association analysis, we first used the AD GWAS summary statistics to directly evaluate the association of rs1990622 variant with AD in IGAP and UK Biobank. We extract the corresponding summary statistics of rs1990622 variant including the beta coefficient (effect size) and standard error in these datasets, respectively. We then determine the heterogeneity of rs1990622 variant in both datasets using Cochran’s *Q* test [21–26]. Finally, we conducted a meta-analysis to evaluate the association between rs1990622 variant and AD using R package (meta: General Package for Meta-Analysis) [21–26]. The overall OR is calculated by the fixed effect model (Mantel-Haenszel) or random-effect model (DerSimonian-Laird), which is determined by the heterogeneity [21–26]. Meanwhile, we further perform additional sex stratification analysis only using the UK Biobank GWAS summary datasets. The statistically significant association for heterogeneity test and meta-analysis is defined to be *P* < 0.05.

### Gene expression analysis of *TMEM106B*

In order to evaluate the expression of *TMEM106B* in different human tissues, we conduct a gene expression analysis using the gene expression data in GTEx (version 8, dbGaP Accession phs000424.v8.p2) [27]. GTEx (version 8) consists of 49 tissues, 838 donors, and 15,201 samples with the number of samples with genotype ≥ 70 [27]. Illumina TruSeq RNA sequencing and Affymetrix Human Gene 1.1 ST Expression Array were selected to measure the levels of gene expression, which was quantified by transcripts per million (TPM) based on the GENCODE 26 annotation [27]. Here, *T* test or analysis of variance (ANOVA) was selected to evaluate the potential difference *of TMEM106B* expression in different human tissues. The statistical significance is *P* < 0.05.

### eQTLs analysis of rs1990622 variant

In all these selected eQTLs datasets, an additive model was used to indicate the rs1990622 *genotype dosages* including CC = 0, CT = 1, and TT = 2, and a linear regression analysis was used to conduct the eQTLs analysis. For eQTLs analysis in UKBEC, we first downloaded the *TMEM106B* gene expression data and the rs1990622 genotype data [12]. We then evaluated the association between rs1990622 variant and *TMEM106B* gene expression using a linear regression analysis [12]. For eQTLs analysis in GTEx (version 8), we used the online GTEx eQTL Calculator with the linear regression method to evaluate the association rs1990622 variant and *TMEM106B* expression [13]. In Mayo RNAseq Study, a linear regression was used to perform the eQTLs analysis by correcting for *APOE* ε4 dosage, age at death, gender, and multiple technical variables [20]. Here, we downloaded the summary results from the Mayo RNAseq Study to directly evaluate the association rs1990622 variant and *TMEM106B* expression [20]. The statistically significant association is defined to be *P* < 0.05/27 = 1.85E−03. The suggestive association is defined to be *P* < 0.05.

### Colocalization analysis

In order to provide evidence of the AD GWAS and eQTLs pair influencing both AD and the *TMEM106B* expression at a particular region, we performed a colocalization analysis using Coloc: a package for colocalization analyses [28, 29]. Coloc could test five hypotheses based on the posterior probability (PP) for colocalization: H0—neither trait has a genetic association in the region; H1/H2—only trait 1/trait 2 has a genetic association in the region; H3—both traits are associated, but with different causal variants; and H4—both traits are associated and share a single causal variant [28, 29].

## Results

### Genetic association analysis of rs1990622 with AD

In IGAP, the results showed that rs1990622 T allele was significantly associated with increased AD risk (*P* = 5.42E−03). Interestingly, this finding was successfully replicated in UK Biobank (*P* = 1.20E−02). Importantly, the sex-specific analysis indicated that rs1990622 T allele was only significantly associated with increased AD risk in females (*P* = 5.74E−04), but not in males (*P* = 6.48E−01), as provided in Table 2. In both IGAP and UK Biobank, we found no significant heterogeneity with Cochran’s *Q* test *P* = 0.3833. A meta-analysis using the fixed effect model showed significant association between rs1990622 variant T allele and AD risk (OR = 1.03, 95% CI 1.01–1.05, *P* = 2.00E−04).

**Table 2:** Association between rs1990622 variant T allele and AD

Dataset	Disease	Beta	SE	*P* value
IGAP	AD	0.040	0.014	**5.42E−03**
UK Biobank	AD	0.025	0.010	**1.20E−02**
UK Biobank	AD in females	0.043	0.012	**5.74E−04**
UK Biobank	AD in males	0.008	0.017	6.48E**−**01

*SE* standard error. Beta is the regression coefficient based on the effect allele. Beta > 0 and beta < 0 mean that this effect allele could increase and reduce AD risk, respectively. The statistically significant association is defined to be *P* < 0.05

### Gene expression analysis of *TMEM106B*

**Fig. 1:**
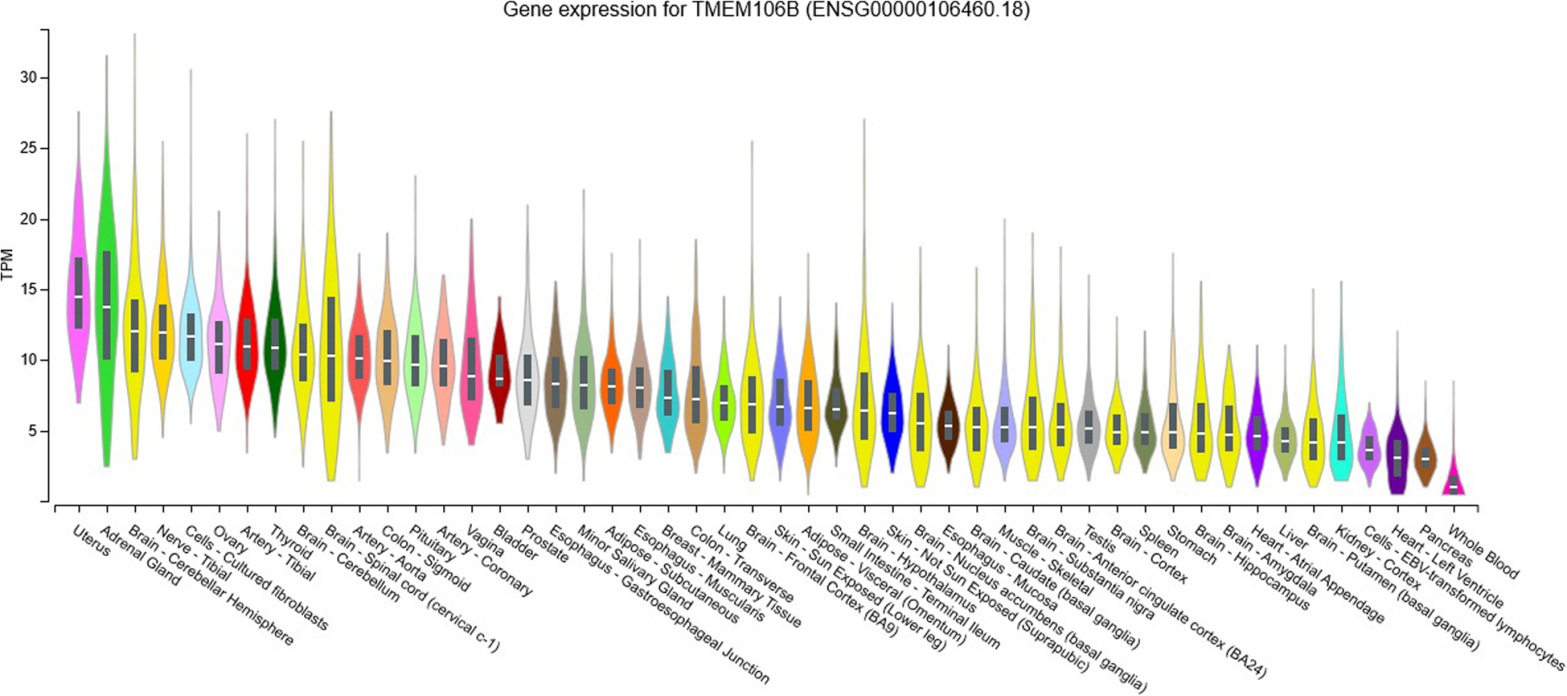
The box plots for the expression of *TMEM106B* in different tissues in GTEx. The gene expression values are shown in transcripts per million (TPM). The gene expression level was quantified by TPM based on the GENCODE 26 annotation, collapsed to a single transcript model for each gene using a custom isoform collapsing procedure [27]

In these selected 49 human tissues from GTEx, the top 10 human tissues with high *TMEM106B* expression are uterus (*n* = 142, TPM median = 14.48), adrenal gland (*n* = 258, TPM median = 13.72), cerebellar hemisphere (*n* = 215, TPM median = 12.02), tibial nerve (*n* = 619, TPM median = 11.90), cultured fibroblast cells (*n* = 504, TPM median = 11.66), ovary (*n* = 180, TPM median = 11.14), tibial artery (*n* = 663, TPM median = 10.94), thyroid (*n* = 653, TPM median = 10.90), cerebellum (*n* = 241, TPM median = 10.36), and spinal cord (*n* = 159, TPM median = 10.32). Meanwhile, *TMEM106B* shows low expression in other 10 human brain tissues including frontal cortex (*n* = 209, TPM median = 6.904), hypothalamus (*n* = 202, TPM median = 6.468), nucleus accumbens (*n* = 246, TPM median = 5.533), caudate (*n* = 246, TPM median = 5.286), substantia nigra (*n* = 139, TPM median = 5.242), anterior cingulate cortex (*n* = 176, TPM median = 5.237), cortex (*n* = 255, TPM median = 4.939), hippocampus (*n* = 197, TPM median = 4.848), amygdala/amygdalae (*n* = 197, TPM median = 4.848), and putamen (*n* = 205, TPM median = 4.213). The box plots about *TMEM106B* gene expression in different tissues are provided in Fig. 1.

### eQTLs analysis

In UKBEC, eQTLs analysis showed that rs1990622 T allele was not significantly associated with *TMEM106B* expression in all these 10 brain regions, as provided in Table 3. In GTEx, eQTLs analysis indicated that rs1990622 T allele was significantly associated with *TMEM106B* expression in cerebellum (*P* = 1.90E−06), cortex (*P* = 2.20E−05), and cerebellar hemisphere (*P* = 1.50E−03). Meanwhile, rs1990622 T allele also showed suggestive association with *TMEM106B* expression in frontal cortex (*P* = 2.20E−02). Importantly, the rs1990622 T allele could only significantly reduce *TMEM106B* expression in these brain tissues, as provided in Table 3. Hence, the eQTLs findings in UKBEC and GTEx from neuropathologically normal individuals did not support the association between rs1990622 T allele and increased *TMEM106B* expression even in the same brain tissue, as reported in ROSMAP by Yang and colleagues.

**Table 3:** Association between rs1990622 variant T allele and *TMEM106B* expression

Dataset	Beta	SE	*P* value	Brain tissue	Number
UKBEC	0.024	0.046	5.96E−01	Cerebellar cortex	134
UKBEC	0.019	0.053	7.20E−01	Frontal cortex	134
UKBEC	0.037	0.054	4.92E−01	Hippocampus	134
UKBEC	6.57E−05	0.044	9.99E−01	Medulla	134
UKBEC	0.015	0.065	8.19E−01	Occipital cortex	134
UKBEC	− 0.003	0.063	9.63E−01	Putamen	134
UKBEC	− 0.025	0.058	6.69E−01	Substantia nigra	134
UKBEC	− 0.030	0.049	5.38E−01	Temporal cortex	134
UKBEC	− 0.019	0.058	7.48E−01	Thalamus	134
UKBEC	0.046	0.052	3.78E−01	Intralobular white matter	134
GTEx	− 0.084	0.065	1.90E−01	Amygdala	129
GTEx	− 0.100	0.059	9.60E−02	Anterior cingulate cortex	147
GTEx	− 0.025	0.033	4.50E−01	Caudate	194
GTEx	− 0.130	0.041	**1.50E−03**	Cerebellar hemisphere	175
GTEx	− 0.150	0.031	**1.90E−06**	Cerebellum	209
GTEx	− 0.140	0.032	**2.20E−05**	Cortex	205
GTEx	− 0.089	0.039	**2.20E−02**	Frontal cortex	175
GTEx	− 0.009	0.037	8.20E**−**01	Hippocampus	165
GTEx	− 0.048	0.044	2.80E**−**01	Hypothalamus	170
GTEx	0.048	0.034	1.60E**−**01	Nucleus accumbens	202
GTEx	− 0.022	0.032	5.00E**−**01	Putamen	170
GTEx	− 0.047	0.062	4.50E**−**01	Spinal cord	126
GTEx	0.100	0.067	1.40E**−**01	Substantia nigra	114
Mayo	− 0.036	–	2.11E**−**01	Cerebellum in AD	186
Mayo	0.052	–	1.18E**−**01	Cerebellum in non-AD	170
Mayo	− 0.001	–	9.61E**−**01	Temporal cortex in AD	191
Mayo	0.031	–	1.75E**−**01	Temporal cortex in non-AD	181

*SE* standard error. Beta is the regression coefficient based on the effect allele. Beta > 0 and beta < 0 mean that this effect allele could increase and reduce gene expression, respectively. The statistically significant association is defined to be *P* < 0.05/27 = 1.85E−03. The suggestive association is defined to be *P* < 0.05

In Mayo datasets, eQTLs analysis indicated no significant association of rs1990622 T allele with TMEM106B expression in cerebellum and temporal cortex (Table 3). Hence, the most significant association between rs1990622 T allele and reduced *TMEM106B* expression identified in neuropathologically normal individuals was not successfully replicated in neurological disease individuals. Here, we provided more detailed results about eQTLs analysis in Table 3.

### Colocalization analysis

**Fig. 2:**
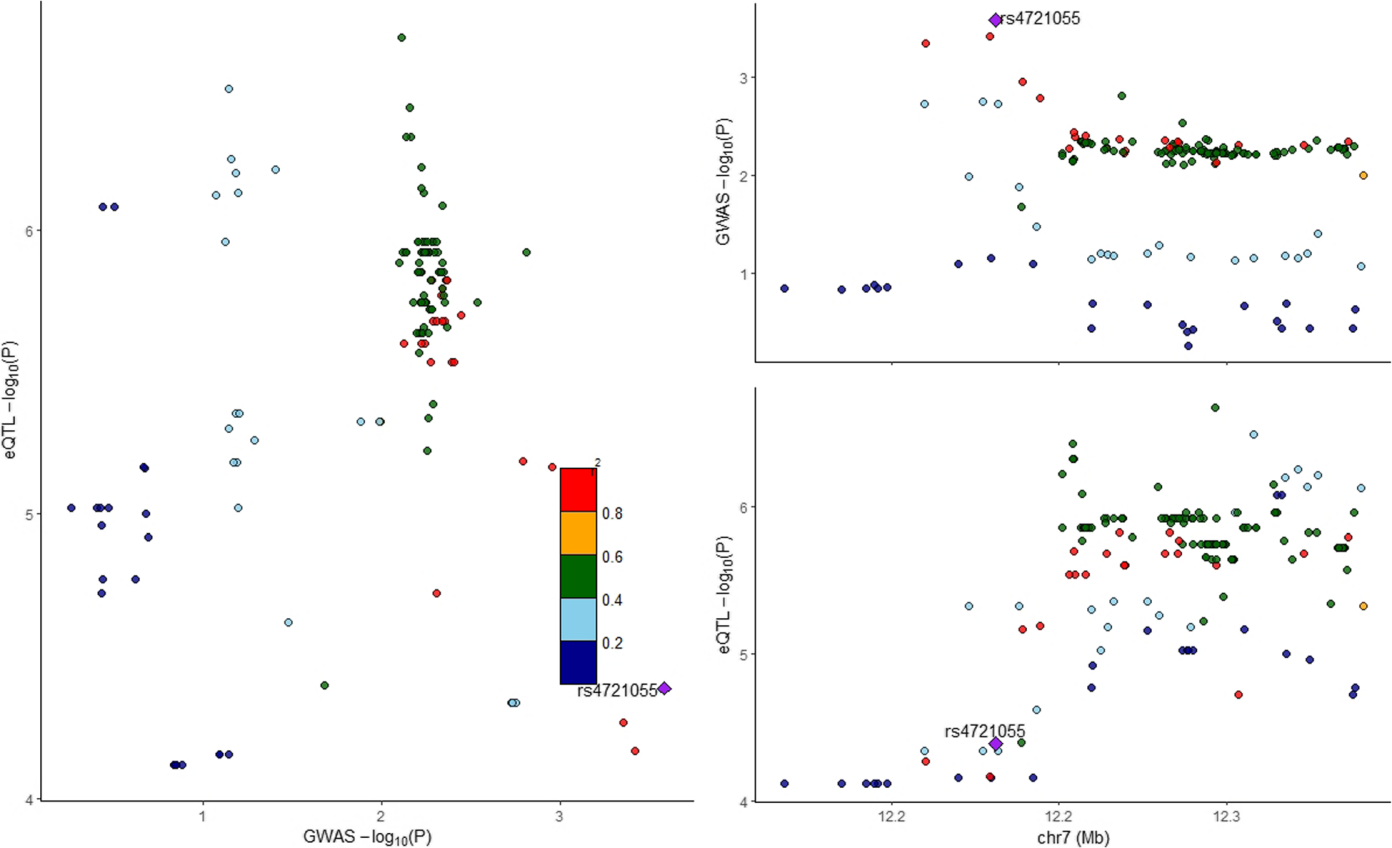
Colocalization analysis of genetic variants associated with *TMEM106B* expression in GTEx cerebellum and AD risk. Created using *locuscomparer* R package. *Coloc* PP0 = 0.031, PP1 = 0.002, PP2 = 0.735, PP3 = 0.037, and PP4 = 0.196. The eQTLs dataset is from GTEx cerebellum (*n* = 209). The AD GWAS dataset is from the IGAP including 21,982 AD and 41,944 cognitively normal controls

In GTEx cerebellum eQTLs dataset, we got 181 genetic variants, which could regulate *TMEM106B* expression with the genome-wide significance level *P* < 1.00E−04. We then integrated these 181 genetic variants with AD GWAS dataset from IGAP using Coloc. The results showed that *TMEM106B* had suggestive evidence (PP4 = 20%) of sharing the same variant with AD risk and *TMEM106B* expression in cerebellum (PP0 = 0.031, PP1 = 0.002, PP2 = 0.735, PP3 = 0.037), as described in Fig. 2.

## Discussion

It has been well established that the *TMEM106B* rs1990622 variant was a FTD risk factor [30, 31]. Until recently, growing evidence highlights the role of *TMEM106B* in other neurological processes including hippocampal sclerosis of aging [32], neuronal loss [31], cognitive deficits [31], better residual cognition [30], AD [9, 33], Parkinson’s disease [34], and amyotrophic lateral sclerosis [34]. Importantly, Yang and colleagues identified the *TMEM106B* rs1990622 variant to show a significant modQTL effect, and highlighted the converging effects of *APOE*-Aβ and *TMEM106B* [5]. However, it remains largely unclear about the role of rs1990622 variant in AD. Here, we conducted comprehensive analyses including genetic association study, gene expression analysis, eQTLs analysis, and colocalization analysis.

Using the genetic association analysis, we evaluated the association of rs1990622 variant with AD using two independent large-scale GWAS datasets from IGAP and UK Biobank, and then conducted a meta-analysis [10, 11]. Interestingly, the results are consistent in both IGAP and UK Biobank, which indicated that rs1990622 was significantly associated with AD risk in both datasets (Table 2). A sex-specific analysis in UK Biobank further indicated that rs1990622 T allele only contributed to increased AD risk in females, but not in males (Table 2). Tropea and colleagues have also evaluated the association of rs1990622 variant with AD using 300 AD cases and 137 neurologically normal control subjects [7]. However, Tropea and colleagues did not identify any significant association of rs1990622 with AD [7]. We think that this may be caused by inadequate sample sizes.

It is known that rs1990622 variant is a non-coding mutation. Hence, it remains unclear how rs1990622 variant affects AD risk. eQTLs analysis is an important method to evaluate the roles of non-coding genetic variants especially the GTEx project, which established a data resource and tissue bank to study the relationship between genetic variation and gene expression in multiple human tissues [35]. To explore the effect of rs1990622 variant in AD risk by regulating *TMEM106B* expression, eQTLs analysis should be conducted in neuropathologically normal individuals or in a general population based on the three considerations. First, eQTLs analysis in AD patients could not be interpreted in terms of AD risk or susceptibility as lack of healthy controls or general individuals [36]. Second, it is well known that disease statuses could change the expression of a specific gene. Hence, gene expression analysis often indicated dysregulated genes in cases compared with controls [37]. Take *TREM2* for example, its expression is upregulated in multiple pathological conditions such as Parkinson’s disease, amyotrophic lateral sclerosis, stroke, traumatic brain injury, and AD, compared with normal controls [37]. Until now, most eQTLs studies focusing genetic variants associated with neurological diseases were conducted in neuropathologically normal individuals, such as AD (UKBEC [38], and GSE15745 [38], and 128 normal subjects [39]), progressive supranuclear palsy (387 normal subjects) [40], schizophrenia (UKBEC [41], GTEx [41], 128 normal subjects [39], and 120 normal subjects [42]), Parkinson’s disease (128 normal subjects [39], GTEx [43]), and bipolar disorder (120 normal subjects [42], GTEx [44], and UKBEC [44]). Meanwhile, other eQTLs studies using both AD and other controls were also reported by adjusting for disease status and some critical covariates [45, 46]. Third, Nicholson and colleagues have reviewed recent findings and found that the significant association between rs1990622 and *TMEM106B* mRNA expression identified in lymphoblast cells could not be successfully replicated in postmortem brain tissues [2]. It is possible that the variable levels of neuronal loss and cell type composition may have masked the association between rs1990622 and *TMEM106B* mRNA expression [2]. Hence, Nicholson and Rademakers suggested that eQTLs studies might be best conducted in non-diseased tissues [2].

Considering these above findings, we then performed an eQTLs analysis to evaluate the effect of rs1990622 variant on *TMEM106B* expression in multiple human brain tissues from neuropathologically normal individuals (UKBEC and GTEx), and further compared the findings from neurological disease individuals (Mayo and ROSMAP). In UKBEC, we found no significant association between rs1990622 variant and *TMEM106B* expression in 10 brain regions, as provided in Table 3. In GTEx, we found that rs1990622 T allele could significantly reduce *TMEM106B* expression in cerebellum (*P* = 1.90E−06), cortex (*P* = 2.20E−05), and cerebellar hemisphere (*P* = 1.50E−03). In Mayo, we found no significant association of rs1990622 with *TMEM106B* expression in cerebellum and temporal cortex. In ROSMAP, Yang and colleagues found that the rs1990622 T allele could increase *TMEM106B* expression in human prefrontal cortex [5]. In summary, rs1990622 variant showed different association with *TMEM106B* expression in neuropathologically normal individuals and neurological disease individuals (Mayo and ROSMAP). The differences were even observed across the neuropathologically normal individuals, such as UKBEC and GTEx, and across the neurological disease individuals, such as Mayo and ROSMAP.

We consider that four reasons may contribute to explain these differences. First, disease statuses may have caused these differences. Satoh and colleagues found that both the mRNA and protein levels of TMEM106B were significantly reduced in AD brains compared control brains [9]. Hence, the different expression of *TMEM106B* may further cause different eQTLs findings in AD and controls. Importantly, eQTLs or cQTL analysis using other *TMEM106B* variants including rs3173615 and rs1990621 further supported our findings [4, 6]. Meanwhile, our and other studies have clearly indicated that eQTLs could vary considerably in different disease statuses [16–19, 47–52]. Second, the mean ages at death in different eQTLs datasets may have driven these differences. The mean ages at death were 55 (UKBEC), 59 (GTEx), 72 or 74 (Mayo), and 88 (ROSMAP), respectively, as provided in Table 1. Nicholson and colleagues explained that the variable levels of neuronal loss and cell type composition may have masked the association between rs1990622 and *TMEM106B* mRNA expression in the older population [2]. Third, the percents of females in different eQTLs datasets may have driven these differences. The percents of females were 26% (UKBEC), 33% (donors), 36–53% (Mayo), and 62% (ROSMAP), respectively, as provided in Table 1. This explanation was supported by our genetic association findings that rs1990622 T allele only contributed to increased AD risk in females, but not in males (Table 2). Importantly, recent findings from GTEx also highlighted the impact of sex on gene expression across human tissues [53]. Fourth, the different descents of the selected donors may also have contributed to these differences. The donors in UKBEC, Mayo, and ROSMAP were of European descent. However, about 85.3% donors were of European descent, and others 14.7% were of African, Asian, and Hispanic or Latino descents, as provided in Table 1.

Using the gene expression analysis, we showed that *TMEM106B* had high expression in cerebellar hemisphere, tibial nerve, cerebellum, and spinal cord, but low expression in other 10 human brain tissues including frontal cortex, hypothalamus, nucleus accumbens, caudate, substantia nigra, anterior cingulate cortex, cortex, hippocampus, amygdala/amygdalae, and putamen. Hence, these findings may explain the significant eQTLs results in cerebellum. Importantly, the colocalization analysis further provided suggestive evidence of sharing the same variant with AD risk and *TMEM106B* expression in cerebellum.

We also realized some limitations in our study, although these above findings. First, we only conducted a sex stratification genetic association analysis using the UK Biobank GWAS summary datasets. The sex stratification datasets in IGAP are not publicly available. Meanwhile, the original GWAS genotype datasets from IGAP and UK Biobank are not publicly available, or a long time is needed to request. Hence, we could not determine the interaction between the sex and rs1990622 genotypes using the raw data. Second, our genetic association analysis identified the female-specific role of rs1990622 in AD risk, but female- or male-specific eQTLs datasets are not publicly available. Third, we performed the eQTLs analysis to investigate the role of rs1990622 variant. In fact, the modQTL analysis may also be important, as did by Yang and colleagues. However, the original gene expression datasets limit our further modQTL analysis. Hence, we will further conduct additional sex stratification analysis, female-specific eQTLs analysis, and modQTL analysis when all these datasets are publicly available.

## Conclusions

Here, we performed comprehensive analyses and found (1) FTD risk variant rs1990622 contributed to AD risk. This cross-disease approach may delineate disease-specific and common features, which will be important for both diagnostic and therapeutic development purposes; (2) *TMEM106B* showed different expression in different human brain tissues especially high expression in cerebellum; (3) rs1990622 variant could regulate the expression of *TMEM106B* in human brain tissues, which vary considerably in different disease statuses, the mean ages at death, the percents of females, and the different descents of the selected donors; (4) colocalization analysis provided suggestive evidence that the same variant contributed to AD risk and *TMEM106B* expression in cerebellum. These findings highlight the importance to better understand *TMEM106B* function and dysfunction in the context of normal aging and neurodegenerative diseases.

## Data Availability

All relevant data are within the paper. The authors confirm that all data underlying the findings are either fully available without restriction through consortia websites, or may be made available from consortia upon request. UK Biobank: http://www.ukbiobank.ac.uk/scientists-3/genetic-data/; https://www.ccace.ed.ac.uk/node/335; IGAP consortium data are available at http://web.pasteur-lille.fr/en/recherche/u744/igap/igap_download.php; https://www.niagads.org/datasets/ng00075; eQTLs in UKBEC: http://www.braineac.org/; eQTLs in GTEx: http://www.braineac.org/; eQTLs in Mayo: https://www.niagads.org/datasets/ng00025.

## Abbreviations

AD: Alzheimer’s disease
cQTL: Cell type quantitative trait loci
eQTLs: Expression quantitative trait loci
GWAS: Genome-wide association study
modQTL: Module quantitative trait loci
